# Machine Learning-Assisted Approaches in Modernized Plant Breeding Programs

**DOI:** 10.3390/genes14040777

**Published:** 2023-03-23

**Authors:** Mohsen Yoosefzadeh Najafabadi, Mohsen Hesami, Milad Eskandari

**Affiliations:** Department of Plant Agriculture, University of Guelph, Guelph, ON N1G 2W1, Canada; myoosefz@uoguelph.ca (M.Y.N.);

**Keywords:** artificial intelligence, bigdata, complex traits, data-integration strategies, deep learning, ensemble learning, random forest

## Abstract

In the face of a growing global population, plant breeding is being used as a sustainable tool for increasing food security. A wide range of high-throughput omics technologies have been developed and used in plant breeding to accelerate crop improvement and develop new varieties with higher yield performance and greater resilience to climate changes, pests, and diseases. With the use of these new advanced technologies, large amounts of data have been generated on the genetic architecture of plants, which can be exploited for manipulating the key characteristics of plants that are important for crop improvement. Therefore, plant breeders have relied on high-performance computing, bioinformatics tools, and artificial intelligence (AI), such as machine-learning (ML) methods, to efficiently analyze this vast amount of complex data. The use of bigdata coupled with ML in plant breeding has the potential to revolutionize the field and increase food security. In this review, some of the challenges of this method along with some of the opportunities it can create will be discussed. In particular, we provide information about the basis of bigdata, AI, ML, and their related sub-groups. In addition, the bases and functions of some learning algorithms that are commonly used in plant breeding, three common data integration strategies for the better integration of different breeding datasets using appropriate learning algorithms, and future prospects for the application of novel algorithms in plant breeding will be discussed. The use of ML algorithms in plant breeding will equip breeders with efficient and effective tools to accelerate the development of new plant varieties and improve the efficiency of the breeding process, which are important for tackling some of the challenges facing agriculture in the era of climate change.

## 1. Introduction

The global population is growing exponentially, and this growth is placing increasing pressure on the world’s resources [1]. The consequences of this exponential population growth are severe, including the depletion of natural resources, the destruction of ecosystems, and the exacerbation of poverty and inequality [2]. The current population of 8 billion is already having detrimental effects on the planet, and, with the population expected to reach 9.7 billion by 2050, these effects will only become more severe [1]. One of the most concerning challenges that this population growth poses is ensuring that there is enough food for everyone. The UN estimates that by 2050, global food demand will increase by 60% compared to what it was in 2010 [3]. Plant breeding, as one of the promising strategies for improving food security in the near future, is known as a powerful tool for reducing hunger worldwide. In recent years, plant breeding has been used to develop new varieties of crops that are more resilient to environmental changes, pests, and diseases and can produce higher yields [4,5], which in turn has resulted in increasing food production and more productive farming systems.

Plant breeding has come a long way from its earliest days. In recent decades, plant breeding has undergone fundamental advancements, with many new technologies and techniques, such as hybridization, genetic engineering, and molecular marker-based breeding strategies, being developed to facilitate the development of superior varieties [6,7]. In order to efficiently implement these techniques in plant breeding programs, plant breeders need to collect and analyze a wide range of datasets, such as omics, for their target traits. The application of omics in plant breeding has revolutionized the field of agricultural biotechnology [8], which makes omics technologies the go-to tool for plant breeders seeking to improve yield [9], increase stress tolerance [10], and develop new varieties of crops [11]. Omics technologies refer to a group of interdisciplinary methods that involve the analysis of large datasets generated from research in different omics fields such as phenomics, genomics, transcriptomics, proteomics, and metabolomics [10]. By examining the genetic makeup of plants, scientists try to identify traits that are beneficial for crop production and then select for those traits in breeding programs [11]. For instance, genetic sequencing of plants has allowed researchers to identify genes associated with important traits, such as disease resistance. By selectively breeding plants with these desirable traits, breeders can create new varieties of crops that are better suited to a particular environment [10]. Another promising application of omics technologies in plant breeding is the use of gene-editing techniques such as CRISPR-Cas9 [12,13]. This technology allows scientists to precisely “edit” the genes in plants, enabling them to introduce beneficial characteristics into the genome without the need for expensive and time-consuming traditional breeding approaches. Lastly, omics technologies can be used to identify and select gene combinations that confer protective benefits [14]. For instance, scientists have used transcriptomics to identify gene combinations that confer resistance to pests and diseases, allowing breeders to develop plants better suited to particular stressed environments [15]. Overall, the application of omics technologies in plant breeding has enabled breeders to develop improved varieties of crops that are more productive, nutritious, and resilient. As this technology continues to improve, it is likely to have an even greater impact on crop production in the coming years.

With the increasing availability of omics and other high-throughput technologies, plant breeding produces larger and more complex datasets requiring sophisticated computational methods and algorithms for better analysis and interpretation. High-performance computing, bioinformatics tools, artificial intelligence (AI), and machine-learning (ML) methods are being used to analyze complex data, allowing breeders to extract meaningful insights from their data, develop more efficient breeding strategies, and better understand the genetic bases of plant traits [16]. ML is a subset of AI that allows computers to learn from data without being explicitly programmed. It is used in a variety of areas, from medicine to finance, and is now being embraced by the agricultural industry to improve plant breeding and cultivar development [17]. By leveraging ML algorithms, plant breeders can precisely identify the key traits and characteristics of plants that are most beneficial for their particular needs. As a result, breeders can more quickly develop new crop varieties better suited to their environment and more resistant to potential threats. The integration of ML in plant breeding programs is a growing trend in the agricultural industry, providing more cost-effective and efficient strategies for breeding new varieties of crops [16].

While the potential benefits of using ML in plant breeding are enormous, some challenges need to be addressed before it can be widely integrated into breeding programs. ML algorithms require large, high-quality datasets and, therefore, significant computing resources [18]. ML algorithms, to some extent, are difficult to understand and interpret [18,19], making it difficult for breeders, who are not necessarily experts in ML and large data analysis techniques, to trust the results and adjust their breeding strategies accordingly. In addition, selecting the most appropriate ML algorithms for a given breeding program and trait of interest is still controversial and challenging for many breeders who are reluctant to use and integrate ML algorithms into their breeding programs. Different ML algorithms have different strengths and shortcomings, and often no single algorithm is suitable for all situations. In this review, we try to (1) define bigdata and review its bases, (2) briefly explain the bases of AI, ML, and their related sub-groups, (3) elaborately explain the bases and functions of some learning algorithms that have been frequently used in plant breeding, (4) review recent advances in the integration of ML in plant breeding programs, (5) briefly explain the three common data integration strategies suitable for combining different breeding datasets using various learning algorithms, (6) review recent advances in the integration of ML in plant breeding programs, and (7) provide future prospects for using novel algorithms in plant breeding. The ultimate goal of the current review is to provide a comprehensive overview of the use of bigdata and ML in plant breeding and to discuss the potential challenges and opportunities associated with their use in this field.

## 2. What Is Bigdata in Plant Breeding?

**Figure 1:**
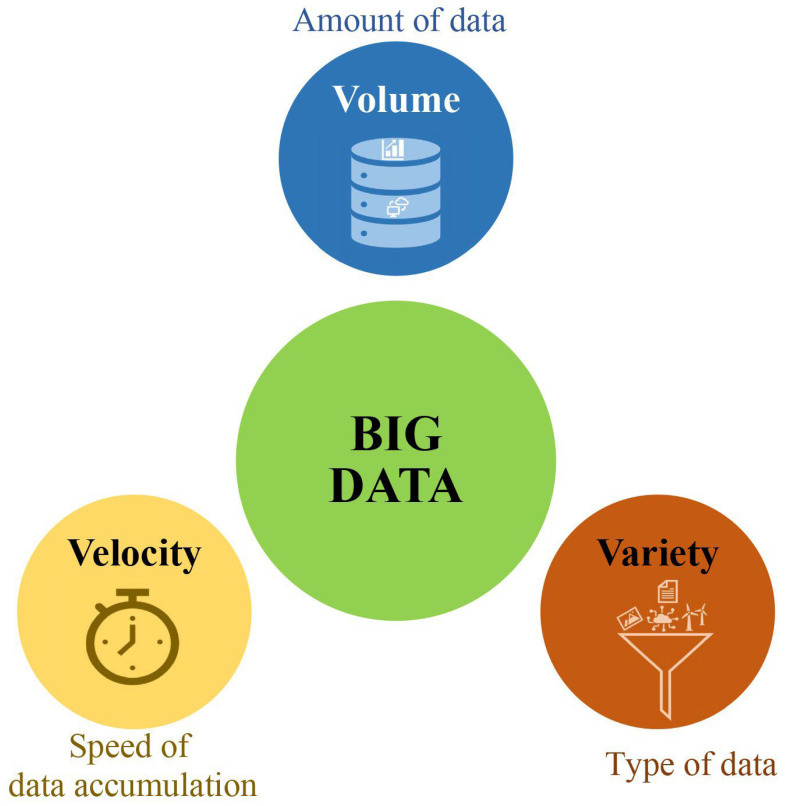
A schematic representation of the three Vs of bigdata. This figure was created using Excel, Microsoft Office 2021.

Bigdata is a term used to describe massive volumes of structured and unstructured data that are difficult to process using traditional statistical approaches [20]. Bigdata is commonly defined by three main Vs: volume, velocity, and variety (Figure 1) [21].

Volume refers to the sheer amount of data that should be processed in a research project or program, usually expressed in terms of bytes, kilobytes, megabytes, gigabytes, terabytes, etc. [22]. In the context of bigdata, velocity refers to the speed at which the data is generated and should be processed to provide useful insights [23]. Velocity is a key factor in determining the effectiveness of data analyses and the efficiency of data processing. As a rule of thumb, the faster data are processed, the faster insights can be obtained from the data. Data processing at a high velocity also allows for a more timely analysis of the data, which can be used for decision making [23]. Variety refers to the diversity of data that needs to be processed, which can come in a wide range of formats, including structured (e.g., numerical data) and unstructured (e.g., texts and images) data [22].

Bigdata in plant breeding is the use of high-throughput technologies, such as omics, to collect and analyze large volumes of data on plant characteristics and their interactions with the environment [24]. This data is used to identify and improve desirable traits, such as plant yield. Volume in plant breeding can include genetic analysis results, growth observations, and environmental data [25]. By tracking the volume of data produced, breeders can identify the experiments that are the most productive and the areas of a breeding process that require additional data [26]. Velocity in breeding datasets can help researchers identify how quickly new data are being produced with different environmental conditions, germplasms, and breeding materials, and if the rate of data generation is fast enough to keep up with the pace of the breeding process [27]. By monitoring the velocity of data generation, breeders can adjust their experiments or increase their data collection efforts as needed [28]. Breeders often deal with a variety of datasets, ranging from structured to unstructured datasets. Genomics datasets can be considered structured datasets as the data are organized in columns and rows, and the information is stored in a tabular format. On the other hand, unstructured datasets in plant breeding may include field notes, images, photographs, and videos. These datasets are not necessarily organized in columns and rows and are more difficult to analyze. However, they can reveal valuable insights into a plant’s behavior and characteristics, which can help breeders select promising genotypes. For example, a researcher may take notes and pictures to better understand how a plant variety performs in different environmental conditions. Similarly, photographs and videos can be used to observe a plant’s physical characteristics and their responses to environmental stimuli.

In addition, bigdata in plant breeding can uncover new insights into the biology of plants, such as genetic markers associated with specific traits, the yield of a particular crop under different growing conditions, or the genes that can be used to develop new varieties with specific characteristics [24]. The growth of bigdata has enabled plant breeders to study interaction effects in more detail using online resources. For instance, DNA–DNA and DNA–RNA interactions, which are very informative in plant breeding and cultivar development, can be found in sources such as WheatExp [29], ZEAMAP [30], and RiceData [31]. Additionally, the exploration of transcription-factor (TF)–DNA interactions should be given great attention in plant breeding in the near future. Different TF–DNA databases, such as PlantPAN [32], JASPAR [33], and PlnTFDB [34], are available for detecting TF binding sites in plants. Moreover, there are more than 100 databases that illustrate and integrate protein–protein interaction (PPI) networks, such as the Predicted Rice Interactome Network (PRIN) and the Protein–Protein Interaction Database for Maize (PPIM) [35,36]. Lastly, metabolic interaction databases such as AraCyc [37], KEGG [38], and PlantCyc [39] provide validated information on biological pathways in plants. As these databases are projected to be improved in the upcoming years, they will play a key role in omics analysis [31].

One of the key steps in the use of bigdata in plant breeding is to efficiently analyze the data using appropriate statistical and mathematical approaches, including statistical techniques, AI, and ML algorithms [40]. By using appropriate data-driven approaches, plant breeders can make better decisions about which “idiotype” varieties to breed and which crops to cultivate. This will ultimately lead to more efficient and successful crop production systems, which will affect food security and prevent global poverty. Some new analysis approaches are explained in the following sections.

## 3. Artificial Intelligence at a Glance

Artificial Intelligence (AI) is the science of making machines and computer systems “think and act like humans” [41]. AI has been gaining traction in recent years and is being used in many different fields and industries, from human sciences and healthcare to plant and animal sciences [41,42,43]. AI can be used to automate tasks, identify patterns in large datasets, provide insights, and even make predictions [42]. In plant breeding, AI technologies such as ML, robotics, and computer vision can be used to analyze large amounts of data and identify patterns that can aid in selecting superior plant varieties with desired characteristics [44]. Machine learning is a subfield of AI that focuses on developing algorithms that can learn from data, identify hidden patterns, and make decisions [19]. Machine-learning algorithms can be used to build predictive models, recognize objects in images, and control robotic systems [17,45]. Computer vision is another field of AI that focuses on developing algorithms that can recognize and identify objects in images and videos [46]. Computer-vision algorithms can be used for tasks such as facial recognition, object tracking, and image classification [47]. Robotics in AI focuses on the development of autonomous robots [48]. Robotics is essential for AI applications that require physical tasks, such as assembling parts, navigating spaces, or manipulating objects [49].

Robotics and computer vision have large applications in plant breeding, enabling plant breeders to accurately monitor crop growth and progress in development as well as identify and select plants with desirable characteristics [44,50]. So many sensors, robots, and pipelines have been recently developed that the explanation of their capabilities is beyond the scope of this review paper; however, to give an example, the BoniRob V2 robot was designed and developed from its early version (BoniRob) as a crop scout robot, capable of measuring soil properties, characterizing plants based on their morphological and spectral characteristics using multi-sensor applications, and providing a camera-based solution for controlling weeds using local chemicals [51]. Thorvald II is another successful robot designed to enable high-quality customization for a variety of environments, such as open fields, greenhouses, and polytunnels. This allows for the quick application of the robot for the given environment [52]. The successful use of computer vision and robotics in soybean-root phenotyping was reported by Falk et al. [53]. ARIA 2.0 was developed in their study to allow post-image-capture automation from soybean roots and eliminate image-preprocessing steps, such as image cropping [53].

## 4. Machine Learning: Basis and Function

Machine learning, as an important subfield of AI, has been widely used in different aspects of our lives, such as communication and agriculture, among many others [53,54]. In agriculture, ML algorithms can be used for crop-yield prediction, crop-growth monitoring, precision agriculture, and automated irrigation [8]. ML algorithms are typically divided into three subgroups: supervised learning, unsupervised learning, and reinforcement learning, which are extensively reviewed in Hesami et al. [14]; therefore, we provide only a brief explanation of these subgroups in this review. In supervised learning, the algorithm is trained on a labeled dataset to make predictions based on the data [55]. The model learns by being given a set of inputs and associated outputs and then adjusting its internal parameters to produce the desired output. Supervised learning is the most common subgroup of ML algorithms that are frequently used in plant breeding to predict complex traits in an early growth stage [56], detect genomic regions associated with a specific trait [40], and select superior genotypes via genomic selection [4].

Unsupervised learning is used when data is not labeled, and the algorithm uses the data to find patterns and similarities in the dataset on its own [57]. The model learns by identifying patterns in the data, such as clusters or groups. In plant breeding, unsupervised learning is usually implemented to find possible associations among genotypes within a breeding population, design kinship matrices, and categorize unstructured datasets [57]. Reinforcement learning is another ML algorithm, in which the model is exposed to an environment and receives feedback in the form of rewards or penalties based on its actions [58]. The model learns by taking actions and adjusting its parameters to maximize the total rewards received. Reinforcement learning is quite a new area in plant breeding, and its applications need to be explored more.

Several important factors need to be taken into account for the successful use of ML algorithms in predicting a given complex trait. Factors include, but are not limited to, data collection, pre-processing, feature extraction, model training, model evaluation, hyperparameter tuning, model deployment, and model monitoring [59,60]. These factors are intensively reviewed in several studies and review papers [59,60,61]. In brief, (1) data collection is the process of gathering data from different sources (environments, genotypes, etc.) in different formats, such as images, text, numerical/categorial datasets, or video, for use in model training [28]; (2) the pre-processing step is defined as the cleaning, transforming, and organizing of data to make it more suitable for ML algorithms [59]; (3) feature extraction is the process in which features/variables are extracted from the data to be represented in a form that is more suitable for ML algorithms [18]; (4) model training uses different ML algorithms to fit models to the data [40]; (5) model evaluation is the process of assessing the accuracy and errors of the algorithm against unseen data [27]; (6) the hyperparameter tuning step contains a second round of adjusting the parameters of tested ML algorithms to achieve the best performance [14,45]; (7) model deployment is summarized as the process of deploying a developed model in production, usually in the form of an application [61]; and (8) model monitoring is the process of tracking model performance over time to ensure it remains accurate [40].

In plant breeding, data collection is an essential step involving the collection of data for target traits from a wide range of environments, trials, and plant populations. Plant breeders often work in different environmental settings in order to gain an accurate understanding of the genotype-by-environment interaction in different trials within each environment. Additionally, they measure different traits in order to establish accurate multi-trait breeding strategies, such as tandem selection, independent culling levels, and selection index. As such, any collected data must be precise, accurate, and pre-processed using various packages and software in order to be suitable for plant breeding programs. Recently, the AllInOne R-shiny package was introduced as an open-source, breeder-friendly, analytical R package for pre-processing phenotypic data [62]. The basis of AllInOne is to utilize various R packages and develop a pipeline for pre-processing the phenotypic datasets in an accurate, easy, and timely manner without any coding skills required. A brief introduction to AllInOne is available at https://github.com/MohsenYN/AllInOne/wiki (accessed on 15 February 2023). Feature extraction is another critical step in determining the most relevant variables for further analysis. The recursive feature elimination of 250 spectral properties of a soybean population revealed a significance of 395 nm, in addition to four other bands in the blue, green, red, and near-infrared regions, in predicting soybean yield [63]. This spectral band can be used to complement other important bands to enhance the accuracy of soybean-yield prediction at an early stage. Furthermore, another study investigated the potential of 34 commonly used spectral indices in anticipating the soybean yield and biomass of a Canadian soybean panel, in which the Normalized Difference Vegetation Index (NDVI) was identified as the most pivotal index in predicting soybean yield and biomass concurrently [56].

Plant breeding involves a series of tasks and data analyses that are carried out over multiple years, and, therefore, repeatability and reproducibility are two important factors to consider when establishing a plant breeding program. Plant breeders may be reluctant to use sophisticated algorithms, such as ML algorithms, for analyzing their trials because of the ambiguity regarding whether or not the results will be reproducible and repeatable. Therefore, it is of the utmost importance to ensure proper model training and evaluation and hyperparameter tuning, deployment, and monitoring when we develop an algorithm. To further improve model training in plant breeding, larger datasets from different locations and years, as well as plant populations with different genetic backgrounds, should be collected [64]. Automated tuning methods can be used to optimize hyperparameters in plant breeding datasets. As an example, grid search is a popular automated tuning method, which is based on an exhaustive search for optimal parameter values [65]. Grid search works by training and evaluating a model for each combination of parameter values specified in a grid. It then selects the combination with the best results [65]. Bayesian optimization is another automated tuning method that uses Bayesian probability theory to determine the best set of parameters for a given problem [66]. Bayesian optimization works by constructing a probabilistic model of an objective function based on previously evaluated values. This model is then used to predict the optimal set of parameters for the given problem [66]. It then evaluates the performance of the system with the predicted parameters and updates the model with new information. This process is repeated to maximally optimize the model’s performance for the given dataset. Bayesian optimization is useful for optimizing complex problems with many variables or where the cost of evaluating the objective function is high [66]. As plant breeders work with different omics datasets, all of which are categorized as bigdata context, the developed algorithm can be exploited in cloud-based services such as the Google Cloud Platform to deploy models at scale [67]. To ensure optimal performance, model performance should be monitored over time and analyzed with metrics such as accuracy and precision, along with anomaly detection, to identify areas of improvement [68].

There are other components/methods that are important in reducing possible errors and increasing the ultimate accuracy of ML algorithms, including transfer learning, feature engineering, dimensionality reduction, and ensemble learning. Transfer learning is an ML technique in which a pre-trained model for a task is reused as the starting point for a model on a second task [69]. Transfer learning reduces the amount of data and computation needed to train a model, and it is particularly helpful for improving the model’s performance when the amount of training data for the second task is small [69]. Feature engineering is the process of using domain knowledge of the data to create features (variables) for the ML pipeline. Feature engineering is an informal topic, but it is considered essential in applied machine learning [70]. Feature engineering can help increase the accuracy of machine-learning models by creating features from raw data that help the model learn more effectively and accurately. Dimensionality reduction is the process of reducing the number of random variables under consideration by obtaining a set of principal variables [71]. It can be divided into feature selection and feature extraction. Feature selection is the process of selecting a subset of relevant features for use in model construction [9]. Feature extraction is the process of combining or transforming existing features into more informative representations that are more useful for a given task [72]. Ensemble learning is an ML technique that combines multiple models to create more powerful and accurate models. Ensemble learning is used to improve the accuracy and robustness of ML models [9]. It combines multiple weak learners to form a strong learner that can make more accurate predictions than the single model. The most common ensemble-learning techniques are the bagging, boosting, and stacking algorithms [9].

## 5. Machine Learning: Model Evaluation

One of the most important steps in exploiting ML algorithms for analyzing datasets is to assess how well an ML algorithm performs on a given dataset [19]. The evaluation process starts with the choice of splitting datasets into training, validation, and testing datasets using the following methods: (1) train/test splitting, which involves splitting the dataset into two parts, one for training and one for testing [19], in which models are trained on the training set and then evaluated on the test set; (2) cross-validation, which involves splitting the dataset into *k* subsets and then training and evaluating the model *k* times [73], and, each time, using one of the *k* subsets as the test set and the other *k*-1 subsets as training sets [73]; (3) the bootstrap method, which includes randomly sampling with replacement from the original dataset, and then training and evaluating the model on the generated samples [74]; and (4) leave-one-out cross-validation (LOOCV), which includes splitting the dataset into *n* folds and then training and evaluating the model *n* times [75], and, each time, using one data point as the test set and the other *n*-1 data points as training sets [75].

The choice of splitting datasets in plant breeding completely depends on the nature of the datasets. Train/test splitting is useful when you have a large amount of data and computational resources are not a concern [57]. This method is particularly useful for data such as genetic markers and RNA sequences, which often possess a large number of data points. By dividing the original dataset into separate training and testing sets, the potential for bias is minimized, and each set will certainly have a wide range of samples [57,71]. Cross-validation, especially *k*-fold cross-validation, is a more robust method for evaluating model performance as it provides an estimate of performance that is less dependent on the specific random train-test split [27]. It is useful when we deal with datasets containing a smaller amount of data (e.g., the development of phenotypic algorithms) or want to ensure that the model is not overfitted towards a specific train/test split. LOOCV is a specific form of cross-validation where the size of the validation set is set to one, and the model is trained on the remaining observations [76]. This method is useful when the dataset is small and has limited data points. LOOCV can be used in plant tissue culture, one-location trials, or greenhouse experiment datasets with a small number of genotypes [76,77].

In terms of measuring the performance of ML methods, there are several error evaluation methods that can be used to measure the overall performance of ML algorithms for their prediction accuracies. Selecting an appropriate error evaluation method depends on the type of outputs: regression or classification. In regression, the mean squared error (*MSE*), the root mean squared error (*RMSE*), the mean absolute error (*MAE*), the coefficient of determination (*R*^2^), and the Akaike information criterion (*AIC*) are the most common error evaluation methods. *MSE* is a measure of the difference between predicted values and true values [78]. It is commonly used as a loss function in regression ML algorithms to measure the accuracy of a model’s predictions [78]. The *MSE* is calculated by taking the average of the squared differences between the predicted estimates and true values. A lower *MSE* indicates a better fit between the model and the data. Equation (1), for calculating *MSE*, is shown below:

(1) $$MSE=\frac{\sum_{i=1}^{n}{\left(Y^{\prime }-Y\right)}^{2}}{n}$$

where *Y′* represents the predicted value, *Y* stands for the observed value, and *n* stands for the total number of observations.

*RMSE* measures how well a regression model fits the data [79]. It is calculated by taking the average of the squared errors between the predicted values and the actual values [79]. It is used as a measure of the accuracy of a regression model, and is calculated using Equation (2):

(2) $$RMSE=\sqrt{\frac{\sum {\left(Y^{\prime }-Y\right)}^{2}}{n}}$$

*R^2^*-squared measures how much of the variance in the data is explained by the model [80]. It ranges from zero to one, with higher values indicating a better fit, and is calculated using Equation (3):

(3) $$R^{2}=\frac{SST-SSE}{SST}$$

where *SST* represents the sum of squares for the total, and *SSE* is the sum of squares for the error.

*MAE* is a measure of the average magnitude of the errors in a set of predictions without considering their direction [79]. It is the average of the absolute differences between prediction and actual observation over the test sample, where all individual differences have equal weight. *MAE* is the most common metric for regression models and is a good general-purpose error metric, and is calculated using Equation (4):

(4) $$MAE=\frac{\sum_{i=1}^{n}\left|{Y^{\prime }}_{i}-YI\right|}{n}$$

*AIC* is used to compare models and evaluate the performance of a regression model [81]. It is calculated by taking into account the number of parameters used in the model, the quality of the fit, and the number of observations [81]. The lower the *AIC*, the better the model. It is calculated by using Equation (5):

(5) AIC=2x−2ln⁡(L)

where *x* stands for the total number of factors, and *L* represents the maximum number of the likelihood function in the algorithm.

In classification models, several methods, such as accuracy, precision, recall, and F1 score, are regularly used to determine how well a model is able to make predictions based on different classes. The accuracy of an ML algorithm is a measure of how well it can predict the classes for a given set of data [82]. This is usually expressed as a percentage and is determined by dividing the number of correct predictions made by the total number of predictions. Using precision, we measure the proportion of true positive predictions divided by the total number of positive predictions. Precision is calculated using Equation (6) [83]. Precision measures how well a model is able to identify instances of a certain class, without mistakenly identifying instances of other classes [82]. Recall in ML algorithms measures a model’s ability to return relevant results for a given query [83]. To calculate recall, the number of relevant results returned by the model is divided by the total number of relevant results, as in Equation (7). Higher values of recall indicate that the model is able to find more of the relevant results than lower values of recall. The F1 score is a measure of the accuracy of a model, which is calculated as the harmonic mean of precision and recall [63,83]. The range of F1 scores is between zero and one, where one is the best possible score. F1 is calculated using Equation (8). F1 scores are often used as a metric to compare the performance of different algorithms or configurations of the same algorithm [83]. Equations (6) through (7) are shown below:

(6) $$Precision=\frac{TP+TN}{TP+FP+TN+FN}$$

(7) $$Recall=\frac{TP}{TP+FN}$$

(8) $$F1\;score=\frac{2\times Precision\times Recall}{Precision+Recall}$$

where *TP* stands for true positive samples, *TN* represents true negative samples, *FP* is false positive samples, and *FN* stands for false negative samples in the confusion matrix.

## 6. Common ML Algorithms in Plant Breeding

During the past decade, different ML algorithms have been frequently used in plant breeding research and programs. When it comes to using ML in plant breeding, each algorithm has its own set of advantages and disadvantages, which are listed in Table 1. As an example, multivariate adaptive regression splines (MARS) was used to attribute the shoot quality, multiplication and leaf color responses of the three strawberry species to their major tissue culture nutrients [84]. The MARS algorithm captured the significant factors and their interactions needed to predict the optimal nutrient levels suitable for the three strawberry species, which can be used as an alternative approach for tissue-culture data analyses [84]. Therefore, it would be the breeders’ choice to select appropriate ML algorithms for their breeding datasets. In this section, the bases of four commonly used ML algorithms in plant breeding (support vector machines, random forests, artificial neural networks, and ensemble learning) are briefly explained, and their overall advantages and disadvantages are highlighted at the end.

### 6.1. Support Vector Machine

**Figure 2:**
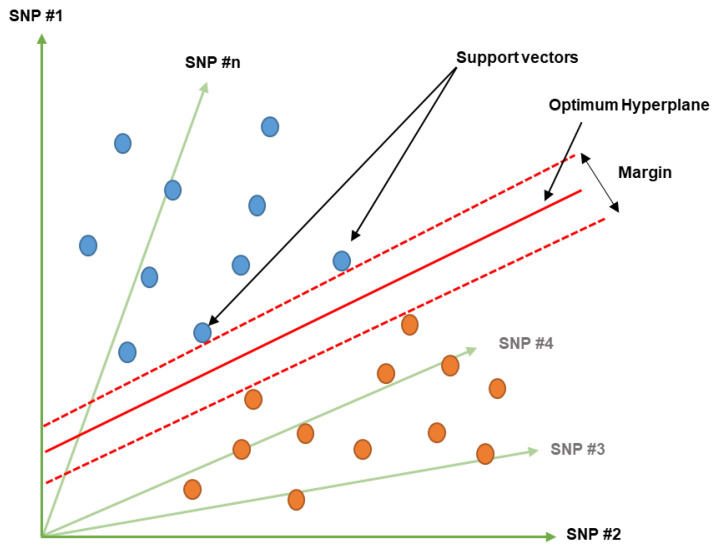
Schematic illustration of a support vector machine used for classifying two sets using SNPs dataset. Red dashed lines illustrate the possible margin among support vectors from two different classes, shown in blue and orange. This figure was created using Excel, Microsoft Office 2021.

Support vector machines (SVMs) belong to supervised machine learning models that are used for classification and regression (support vector regression) problems [85]. SVMs are based on the concept of decision planes that define decision boundaries. A decision plane is one that separates a set of objects that have different class instances (Figure 2). In SVMs, a set of points in a given space are mapped so that the points of different classes are divided by a clear gap that is as wide as possible [85]. The line that separates the classes is the decision boundary. The distance between the decision boundary and the closest point in each class is known as the margin (Figure 2). The points that lie on the margin are called support vectors [86]. The aim of the SVM algorithm is to find the best possible decision boundary or hyperplane that maximizes the margin between the two classes (Figure 2). Support vectors are the crucial elements that help to determine the position and orientation of the hyperplane [85,86].

Zhao et al. [87] investigated the predictive performance of the SVM model using kernel functions and hyperparameters in eight published genomic datasets on pigs and maize. Their results showed that SVM had a higher performance in comparison with the other tested methods in terms of time and memory usage [87]. In another study, Griffel et al. [86] showed that SVM algorithms utilizing near-infrared and shortwave infrared wavelengths can be used to detect potato virus Y (PVY) with an accuracy of 89.8%, outperforming current industry standards based on red, green, and blue wavelengths and traditional computational methods, which yielded an accuracy of only 46.9%. Shafiee et al. [88] investigated the potential use of support vector regression (SVR) with sequential forward selection (SFS) for predicting grain yield in comparison with a LASSO regressor with an internal feature selector using a hyperspectral reflectance dataset. The results showed that SVR had the same performance in comparison with the other tested algorithms but a higher computational burden. Therefore, the LASSO regressor was suggested to analyze hyperspectral reflectance indices [88].

Despite the widespread use of the SVM algorithm in various studies, there is still some controversy surrounding the successful application of SVM in the field of plant breeding, which is rooted in its nature. SVM is effective in high-dimensional spaces, especially in cases where the number of dimensions is greater than the number of samples [59]. It uses a subset of training points in the decision function, so it is also memory efficient. SVM is also versatile, in that different kernel functions can be specified for the decision function [85]. However, SVM does not perform well when the data set has more noise (i.e., target classes are overlapping) [4,59,85]. SVM also cannot directly provide probability estimates, but these can be calculated using an expensive five-fold cross-validation. In the case of having a large, noisy dataset, it is recommended to use other ML algorithms, such as random forests or neural networks [85].

### 6.2. Random Forest

**Figure 3:**
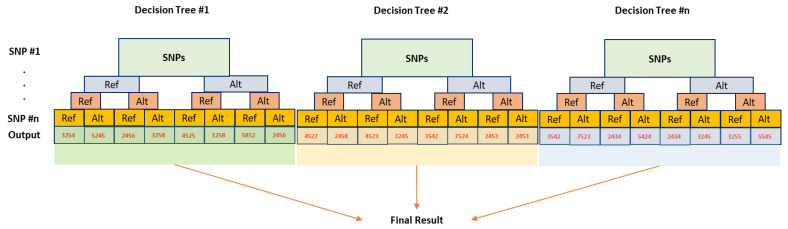
Schematic illustration of random forest used for predicting output (yield as an example) using SNPs dataset. In this case, molecular genetic markers, i.e., SNPs, are input variables, and yield is considered as an output variable. Alternative and reference alleles are expressed as Alt and Ref, respectively. This figure was created using Excel, Microsoft Office 2021.

Random forest (RF) is another common supervised ML algorithm used for both classification and regression. RF combines multiple decision trees to produce a more accurate and stable prediction. The algorithm randomly selects a subset of features from the dataset and then builds multiple decision trees using the features [89]. The predictions from each tree are then combined to form a more accurate prediction than any of the individual trees (Figure 3). RF has the advantage of being able to handle complex datasets with many features, making it a powerful tool for predictive analytics [90].

Parmley et al. [91] reported the successful use of the RF algorithm in predicting soybean seed yield using hyperspectral reflectance bands as the input. A panel of 292 unrelated soybean accessions was studied across six environments to collect data on their phenomic traits and seed yield, and RF was used to analyze the complex relationships among these traits and yields. The results showed the benefit of using RF to identify a set of in-season phenomic traits that allow breeders to reduce their reliance on resource-intensive end-of-season phenotyping, such as seed-yield harvesting. Similar results were confirmed in a study conducted by Yoosefzadeh-Najafabadi et al. [63], in which they examined the effectiveness of three popular ML algorithms (multi-layer perceptrons, SVMs, and RFs) in predicting soybean seed yields using hyperspectral reflectance. The RF algorithm was found to produce the highest yield classification accuracy with a value of 84% and was subsequently used as the best classifier for the ensemble method. The results of this study showed how soybean breeders can employ RF in an ensemble algorithm to efficiently identify high-yielding genotypes from a large number of candidates using either full or selected spectral reflectance at early growth stages. In another study, three decision tree algorithms—chi-squared automatic interaction detector (CHAID), exhaustive CHAID, and classification and regression tree (CART)—were used to predict the effects of minor mineral nutrients on *Corylus avellana* L. cultivars [92]. Of the three algorithms, CART was found to be the most accurate in terms of predicting minor nutrient levels [92]. In an experiment, the effects on plant shoot quality of different levels of mineral nutrients (calcium nitrate, ammonium nitrate, meso compounds, potassium sulfate, and minor nutrients) were tested using a response surface methodology [93]. The data were analyzed using CART, which determined cut-off values for the data and created interpretable data trees [93]. The analysis revealed the efficiency of using CART to select the best shoot quality and growth characteristics of a wild apricot [93]. Aside from the use of RF as a supervised ML algorithm, the possible use of RF in unsupervised learning methods was investigated by Shi and Horvath [94] in a human study. This research has demonstrated that RF dissimilarity is an effective tool for clustering samples (tumors) based on input marker expressions.

The key advantages of RF algorithms are their accuracy, stability, and ability to work well with large datasets [89]. RF can handle a large number of input variables without much tweaking and can identify important variables within a dataset. However, RF algorithms are slow to create predictions once they are fully trained [90]. They are difficult to interpret, meaning they are usually a black-box approach. Additionally, the random selection of features means that its accuracy can vary greatly depending on the data, which makes RF algorithms not well-suited for real-time predictions [89,90].

### 6.3. Artificial Neural Network

**Figure 4:**
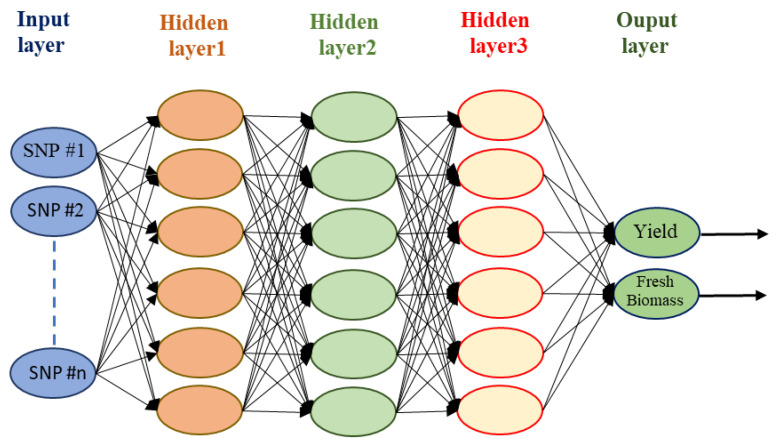
Schematic illustration of an artificial neural network used for predicting two outputs (yield and fresh biomass as an example) using molecular genetic markers, i.e., SNPs, dataset. This figure was created using Excel, Microsoft Office 2021.

An artificial neural network (ANN) is a type of ML algorithm that is modeled after the function of the human brain and can be used to solve complex problems [95]. ANNs are built upon a set of interconnected nodes that mimic the behavior of neurons in the brain [96]. Each node represents a mathematical function that takes a set of inputs and produces an output (Figure 4). The output of one node is sent to other nodes in the network, and the weights of the connections between nodes are adjusted based on the output of the previous node (Figure 4) [95]. ANNs are widely used in ML algorithms because of their ability to learn from the data [96].

Silva et al. [97] proposed a procedure for training networks with expanded data sets and the use of statistical parameters to estimate the breeding values of genotypes in simulated scenarios. In evaluating different artificial neural network configurations, the results showed that the neural network model was superior to linear models for predicting genetic values and had a high generalization performance in validation experiments [97]. The effectiveness of ANN in selection procedures was assessed with data from sugarcane families [98]. The best ANN model tested in this study was proven to be accurate and able to classify all genotypes correctly with no mistakes, thus replicating the selective choice made by the breeder during the simulation of the individual best linear unbiased predictor (BLUP). This highlights the ANN’s capability to successfully learn from training and validation inputs and outputs [98]. The successful use of ANN for evaluating genetic diversity in plants was assessed by Sant’Anna et al. [99], comparing ANN’s accuracy with traditional methods. The results showed that ANN proved effective in classifying populations with both low and high levels of differentiation, even when the datasets had low differentiation [99].

The major advantages of ANNs are their abilities to learn non-linear relationships within a dataset, learn without prior knowledge, handle large amounts of data, and identify patterns and trends in data [95,100]. However, ANNs are usually computationally intensive, and it would be difficult to determine the right architecture. As a result, they are prone to overfitting and local minima [96].

### 6.4. Ensemble Learning

Ensemble learning is an ML technique that combines multiple algorithms to create more powerful models [101,102]. It combines individual predictions of multiple ML algorithms to produce more accurate and robust predictions [103]. Ensemble learning can be used in a variety of ML tasks, such as classification, regression, and clustering. It is particularly useful in cases where individual algorithms may not be as accurate as a combined model [103,104]. The main functions of ensemble learning in ML algorithms include: (1) improving prediction accuracy—by combining multiple models, the performance of the models are improved as they are able to capture different aspects of the data [103]; (2) reducing variance—ensemble learning helps reduce the variance of predictions by averaging out individual models and thus minimizing noise [105]; (3) handling bias–variance trade-off—by combining different models, the bias-variance trade-off is mitigated as the models can capture different aspects of the data [104]; and (4) enhancing interpretability—ensemble learning makes it easier to interpret the results of the individual models as they can be inspected to understand different aspects of the data [103,104]. Ensemble learning is also computationally efficient and fast, as it requires less training data and can be used to train models quickly [105].

**Figure 5:**
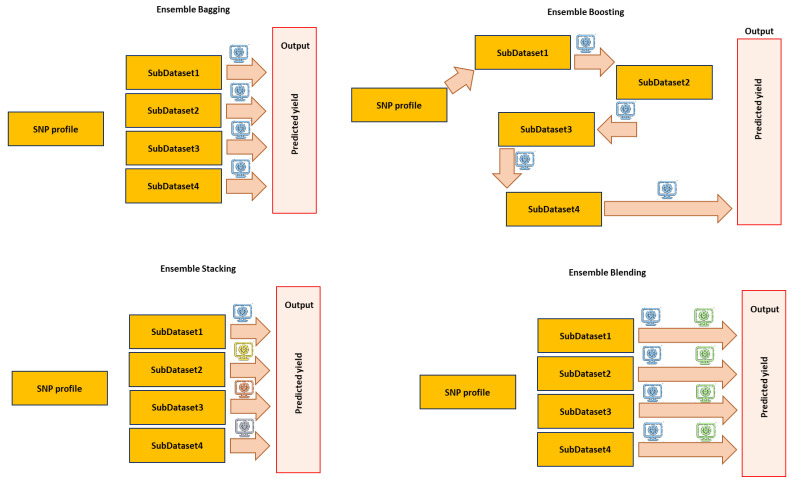
Schematic illustration of different ensemble strategies used for predicting an output (yield as an example) using molecular genetic markers, i.e., SNPs, dataset. Different colors in the computational logos represent different ML algorithms in each ensemble method. This figure was created using Excel, Microsoft Office 2021.

Different types of ensemble learning, such as bagging, boosting, stacking, and blending, are preferred based on different functions and sampling methods. Bagging combines multiple weak learners to create a single strong learner (Figure 5). It uses bootstrap sampling to create multiple datasets from the original dataset and then fits different models to each of the datasets [104]. The final prediction is the average of all the predictions made using the individual models. Boosting ensemble methods work by sequentially adding predictors to an ensemble, each one correcting its predecessor (Figure 5) [105]. In the boosting method, the first predictor is trained on the whole data set, and then each additional predictor is trained on a dataset that contains the mistakes of its predecessors [105]. The predictors are then weighted in order to favor those that are more accurate. The goal is to find the combination of predictors that will yield the best performance. Stacking combines multiple models to generate a single stronger predictive model (Figure 5). The stacking method involves training a base set of models on a training set, predicting the test set with the base models, and then training a higher-level model on the predictions from the base models as inputs [106]. The higher-level model is then used to make final predictions. The main advantage of stacking is that it can combine the strengths of multiple models to generate a more accurate prediction than any of the single models [106]. Stacking can also be used to reduce overfitting, as the higher-level model can act as a regularizer. The blending method is one of the most popular ensemble methods and involves training a second model to learn how to combine the outputs of its constituent models (Figure 5) [107]. The blending model is typically trained using a supervised learning algorithm, such as a decision tree. By combining multiple models in this way, the accuracy of the resulting predictions can be improved [107]; however, it strongly depends on the nature of dataset and applied individual models.

The application of ensemble learning in plant breeding is less studied than the individual ML algorithms. In a cattle-breeding study, an ensemble learning-based genomic prediction model (ELGP) was developed by incorporating eight ML methods for predicting phenotypes from genotypes. The results showed that ELGP outperformed the eight base learners for predicting milk yield, milk fat percentage, and somatic cell score traits [108]. In the plant-breeding field, the use of ensemble algorithms in accelerating the efficiency of genomic-related tools, such as a genome-wide associations study (GWAS) and genomic selection, has been reported previously [109]. One study focused on five key traits contributing to soybean yield, which were measured in a panel of 250 genotypes across 4 environments [110]; multi-layer perceptron, radial basis function (RBF), and RF algorithms were used to predict the yield. RBF was found to be the most accurate algorithm and was selected as the best classifier for an ensemble method based on the bagging strategy (E-B), which improved prediction accuracy up to 10% in comparison with the individual tested algorithms. In addition, by aliening E-B with a genetic algorithm (GA), the researchers modeled the optimal values of yield components in an ideotype soybean with improved yield [110]. In another study, Yoosefzadeh-Najafabadi et al. [4] indicated that the E-B strategy could be used to enhance the accuracy of genome selection for soybeans. Using the E-B strategy and haplotype block datasets rather than full SNP datasets for complex yield component traits can also improve the prediction accuracy of GS by up to 7% [4]. Overall, further research is necessary to assess the efficacy of ensemble algorithms in predicting traits of interest using high throughput phenotyping and genotyping datasets in plant breeding.

## 7. Data Integration Strategy

Data integration strategies in plant breeding are methods that are used to combine data from multiple sources to gain a better understanding of the genetic and environmental factors affecting the success of a breeding program [16]. These strategies can include the integration of data from multiple trials, genotypic and phenotypic data, or data collected using different omics approaches. By successfully integrating different datasets, breeders can gain insights into the interactions among different molecules, pathways, and networks and can have a better understanding of the underlying biological processes of a given characteristic [28]. Additionally, data integration strategies can help identify novel biomarkers and sources of data bias and improve the accuracy of findings [16,28].

A considerable amount of data has been gathered from various plant omics, but the integration of these bigdata in plant breeding has not yet been thoroughly investigated, which necessitates the development and use of multiple data integration approaches. Conceptual, statistical, and model-based strategies are the most common data integration strategies that can be used in plant breeding areas [111]. The conceptual integration strategy is based on the principle of integration, which states that the whole is greater than the sum of its parts [14,112]. It combines data from different sources to gain a more comprehensive understanding of the biological concepts of a trait of interest [111]. The data is then analyzed to identify patterns, relationships, and correlations that can be used to make meaningful biological inferences. To use the conceptual integration strategy, breeders must first identify the datasets relevant to their breeding goals and objectives. They must also consider the quality of the data and the type of analysis that is needed. In the next step, the data must be integrated and analyzed separately, and the breeder must counterpart their results to identify meaningful biological insights. Finally, the results must be validated and interpreted to draw meaningful conclusions.

Statistical integration is a data integration strategy that uses statistical methods to combine multiple data sources into a single, integrated view [113]. It involves applying statistical techniques to identify patterns and correlations among data from different sources and to measure their impact on the overall dataset [111,113]. Statistical approaches include the use of clustering algorithms, which can be used to group similar datasets together and identify patterns in the data, or regression models, which can be used to identify relationships between variables in different datasets [113].

Model-based integration is known as another data integration strategy that uses data-driven models, such as ML algorithms, to represent the relationships between multiple data sources [114]. This strategy is most useful when dealing with highly structured data, as the model can be used to identify complex relationships among data elements [115]. This approach can also be used to help identify data sources that are not linked by any common attributes, allowing for a more comprehensive integration process [114].

Overall, the scale and complexity of breeding datasets pose significant challenges in handling and developing high-performance computational strategies for their applications. To address these challenges, it is important to explore and evaluate different data integration layers to determine which approach is best suited for a particular dataset.

### Recent Advances in Integration Strategies

In the past decade, the use of ML algorithms has been increasingly integrated into various aspects of plant breeding. These algorithms have been used in a variety of ways to improve the efficiency and accuracy of plant breeding practices. One common application of ML algorithms in plant breeding is using predictive analytics for processing images, which involves using aerial or ground-based images to quickly and accurately identify the presence or absence of specific characteristics in plants. This can be performed remotely, using images taken from a distance, or proximally, using images taken up close. Fei et al. [116] explored the possible use of a low-cost, multi-sensor, unmanned aerial vehicle (UAV) data fusion and ensemble learning for grain yield prediction in wheat using five ML algorithms as follows: cubist, SVM, deep neural network, ridge regression, and RF. Based on the results, the multi-sensor data fusion-based yield prediction showed a higher accuracy compared to the individual-sensor data in each ML algorithm [116]. The use of ensemble learning illustrates a further increase in accuracy with *R*^2^ values up to 0.692. Overall, the results proved that low-altitude UAV-based multi-sensor data could be used for early-growth-stage grain yield prediction with high accuracy, using data fusion and an ensemble learning framework. This can improve the efficiency of selection in large breeding activities [116]. In another study, a robust and automatic approach was developed for estimating the relative maturity of soybeans using a time series of UAV images [117]. An end-to-end hybrid model combining convolutional neural networks (CNN) and long short-term memory (LSTM) was proposed to extract features and capture the sequential behavior of time series data [117]. The results suggested that the proposed CNN–LSTM model was more effective than the local regression method. Additionally, the study demonstrated how this newfound information could be used to aid in the advancement of plant breeding. Automated phenotyping is another application of ML in plant breeding, which involves using sensors and other equipment to assess plant traits, such as yield, in an efficient and cost-effective manner [118]. This can be used to identify the most promising lines for further evaluation and to make informed decisions about which traits to breed for [27].

ML algorithms are frequently used in genomic selection to identify the most promising lines for further evaluation. Sandhu et al. [119] investigated the performance of two deep learning algorithms, multi-layer perceptron and the convolutional neural network, in comparison to the widely-used ridge regression best linear unbiased predictor (rrBLUP) model. CV and independent validation, alongside different sets of SNP markers, were employed to optimize the hyperparameters of the DL models, minimizing the root mean square (RMS) in the training set and avoiding overfitting via dropout and regularization. Results showed that the DL models achieved 0 to 5% higher prediction accuracy than rrBLUP for all five traits, with multi-layer perceptron having a further 5% higher accuracy than the convolutional neural network for grain yield and grain protein content. The authors concluded that DL approaches provide better prediction accuracy for each trait, and should be considered for use in large-scale breeding programs [119].

Another area in which the application of ML algorithms is expanding fast is in GWAS and QTL analyses, which involve using genomic data to efficiently detect the genomic regions associated with a trait of interest. This can be used to develop reliable genetic markers for a marker-assisted selection strategy, which can be used to identify the most promising lines for further evaluation in a breeding program. ML-mediated GWAS utilizes advanced algorithms to analyze genetic data and identify single nucleotide polymorphisms (SNPs) associated with a particular trait [120]. These algorithms provide variable importance measurements, which consider the interactions between SNPs and the main effects of individual markers, and are more powerful than conventional GWAS for detecting SNPs with small effects [120,121]. Variable importance methods such as linear and logistic regressions, SVM, and RF are well established in the literature and have been used to further improve ML-mediated GWAS [16,120,122,123,124,125]. Tang et al. [126] have also introduced a new genetic-level importance measure based on scaled variable importance for SNPs, which defines the importance of each SNP as the mean or maximum importance of all SNPs. They recommend using a percentage scale for estimating variable importance and suggest using a global empirical threshold to provide the empirical distribution of a null hypothesis. The number of repetitions and the level of significance (α) should be optimized based on the genetic background, number of markers, distribution of the phenotypic data, and number of data points.

Now, in the era of “Breeding 4.0”, the use of data analytics and technological advancements has allowed researchers to develop sustainable systems globally [127]. This evolution has seen the utilization of prescriptive analytics to make informed decisions. For the success of these initiatives, intentional and standardized data management is necessary to harmonize multidimensional data from an organization and also facilitate community integration for resource sharing [127]. In this era, ML algorithms will remain a key factor in research and product development in agricultural industries [128]. In the upcoming phase, Breeding 5.0, integrated technologies and bigdata will enable crop production systems to determine the best genotype for any given environment [127]. Overall, ML algorithms have been successfully integrated into different aspects of plant breeding and are expected to continue to play an important role in the future of plant breeding.

## 8. Conclusions

ML provides a great opportunity to make plant breeding more efficient and predictable. In this review, we tried to discuss and highlight the main concepts of ML in terms that are relatable to plant breeding and discuss illustrative examples from conventional practices in plant breeding. ML can be employed in almost every step of plant breeding, from selecting appropriate parental lines for crosses to evaluating the performance of advanced breeding lines across several environments. Although this review paper was limited to a discussion on the applications of machine learning in conventional-based plant breeding, high-throughput phenotyping, and genotyping, other breeding-based methods (e.g., in vitro breeding, *Agrobacterium*-mediated genetic transformation, and genome editing) can also benefit from using ML methods. In general, the generation of large, high-quality datasets is a key factor in the continuation of the successful implementation of ML in plant breeding programs. To plan and generate large datasets that can be used for meta-analysis, it is important for the scientific plant breeding community to agree on some fundamental principles, such as data type, content, and data format, to ensure that data generated by different breeders and institutions are compatible and made available, ideally in an ML-readable format. The availability of such datasets can provide a forward-thinking aid to ML-mediated plant breeding and can hopefully overcome many of the challenges faced by plant breeding programs.

## Figures and Tables

**Table 1:** The important attributes of different ML methods in plant breeding based on ranking (low, medium, high, and very high).

ML Method	Hyperparameter Tuning	Overfitting Risk	Explainability	Comparative Accuracy	Complexity	Samples Needed	Computation Cost	Implementation Time
Deep Neural Network	Very high	Low	Low	Very high	Very high	Very high	Very high	Very high
Artificial Neural Network	High	High	Low	High	High	Medium	High	High
Random Forest	High	Medium	High	High	Medium	Low	Medium	Low
Non-linear (Kernel) SVM	High	High	Low	High	High	High	High	High
Linear SVM	High	High	Low	High	Medium	High	Medium	Medium
Decision Tree	Medium	High	High	Medium	Medium	Medium	Medium	Low
Self-Organizing Maps	High	High	Medium	Medium	High	Medium	Low	Low
*K* nearest neighbor	Medium	High	Medium	Medium	High	Medium	Low	Low
Gradient Boosting	Very high	High	Low	High	Very high	Medium	High	Medium
Naive Bayes	Medium	Medium	Medium	Medium	High	Medium	High	Medium
Bayesian Network	High	Medium	Medium	Medium	High	Medium	High	Medium
Partial Linear Regression	Medium	Low	High	Medium	Medium	High	Medium	Low
Logistic Regression	Low	High	High	Low	Medium	Medium	Medium	Low

## Data Availability

Not applicable.
